# Massage in the Treatment of Fractures

**Published:** 1900-02-10

**Authors:** 


### Hospital Clinics and Medical Progress.

### MASSAGE IN THE TREATMENT OF FRACTURES.

It has been held for so long as one of the first principles in the treatment of fractures that, whatever else might be done, the fragments must be kept immovably in apposition, that much interest attaches to the experience of those who of late years have adopted the practice of employing massage and of systematic movement without any retaining apparatus in the treatment of these injuries. Dr. Lucas-Championnière,[1] of Paris, who has for long been well known as an advocate of the latter form of treatment, has recently summarised his conclusion, and from his paper we cull the following details as to his method and its results: He says that both in principle and in practice the system of immobilisation is false and hurtful. Organs of movement were made to be moved; their health and nutrition demand movement; and the absolute suppression of movement, such as is attempted in complete immobilisation, lowers the health of these organs and interferes seriously with the processes of repair. It must be understood, however, that in speaking thus, Dr. Lucas-Championnière is referring to fractures running into joints, and that fracture of the shafts of long bones comes under another category.

In regard then to articular fractures, which include at least a fifth of all the cases of fracture which a surgeon has to treat, it is laid down that moderate movement favours the repair of the fragments. It is admitted that the callus will be greater in quantity, will be more solid, and will be more rapidly thrown out, but this does not seem to be regarded as in any way detrimental. Any large amount of movement would, of course, inhibit the process of repair, or at least seriously interfere with it, but moderate movement preserves the vitality of the limb and the mobility of the joint, the muscles, and the tendons, and entirely prevents the occurrence of muscular atrophy. Hence, unless moderate movement threatens to cause the occurrence of deformity, the limb should not be immobilised.

In addition to passive movement massage may also be applied, "on the single condition that the parts involved in the fracture do not thus become the subject of untimely interference with repair," which seems rather like begging the question. With this proviso we may apply massage immediately after the accident, but it may be noted that the kind of massage which is made use of must be such as can be performed while the fractured member is properly supported. Ordinary massage, or massage administered too actively, would, we are told, certainly lead to lamentable results.

What then is this massage which we are to employ in articular fractures? "The massage for fracture ought to be applied to all parts of the limb, along the muscles, over the ligaments, and upon the hæmorrhagic effusions, but never just over the site of the fracture nor directly over the fractured ends of the bones. The massage ought to consist of gentle, repeated pressure, always in the same direction, from the periphery of the limb towards the trunk. It should be sufficiently prolonged. At least a quarter of an hour to twenty minutes once a day should be devoted to it. The massage should never give pain," and should always be followed by an attempt at passive movement which also should not give pain. When a limb has been massaged and when passive movement has been practised the repair of the fracture is much more rapid than when immobilisation has been used; especially as, when immobilisation apparatus is removed, a second treatment must often be undertaken to overcome the consolidating action, the stiffness and muscular rigidity, produced by the apparatus and by the immobility its use has secured.

By the use of massage and mobilisation alone we can, we are told, treat absolutely without any apparatus the large majority of fractures of the wrists, of the radius, and Colles' fracture. The larger number of fractures of the clavicle are amenable to the same method. "In four years at the Hospital Beaujon," says Dr. Lucas-Championnière, "I have had in my service more than sixty cases of fracture of the clavicle. I have treated all of them without apparatus, using only a simple sling, not once having had occasion to apply any other form of treatment. All the fractures of the upper extremity of the humerus as far down as the insertion of the deltoid may be treated in the same way. All the fractures of the lower extremity of the humerus, all the fractures of the elbow, including those of the olecranon, most of the fractures of the fibula, a large number of the fractures of both malleoli, many of the fractures of the knee, and all of the fractures of the scapula are best treated in this way."

Again, in cases in which this form of treatment cannot be carried out in its entirety, it is recommended that a mixed method be employed, apparatus being applied after a preliminary massage, and removed from time to time in order to allow of the repetition of the process; and in a large number of cases even of fracture of the thigh and the humerus this mixed method is said to be desirable. The same method is also recommended in the secondary treatment of dislocations, after reduction has taken place, and it is stated that it has never led to any danger of recurrence of the displacement.

It is to be noted that while the method recommended is said to give more rapid and satisfactory results than any other in the treatment of fractures, it requires much more time and personal attention on the part of the surgeon than the use of an apparatus which, after being carefully applied, may be left undisturbed for days and even weeks, for this method requires daily inspection and the application of the treatment by someone who is ready and able to give it the most careful attention. What shows that it is being properly done is the complete cessation of pain.

Dr. Lucas-Championnière, however, is by no means alone in the view which he takes as to the desirability of doing away with the prolonged immobilisation which has so long prevailed in the treatment of fractures in the neighbourhood of joints. In 1896 Mr. Noble Smith reported to the Medical Society of London a case of Pott's fracture in which, although splints were applied, almost from the first the ankle was gently moved every day and a gradually increased amount of massage was employed. By the end of the third week the patient could bear weight upon the foot for a moment, on the twenty-third day he could walk with caution, and on the thirty-fourth day he gave up crutches and took to sticks only. Mr. Bennett, again writing in the *Lancet*, in 1898, pointed out that massage in fractures had not received the attention it deserved from English surgeons, and advocated the proceeding, saying that the pain and stiffness which are seen after fractures near joints are due in most cases to the matting together of the soft parts, rather than to adhesions within or about the joints, and pointing out that this might be relieved by massage, which also prevented atrophy of the muscles. But he, like Dr. Lucas-Championnière, admitted that this method of dealing with fractures takes up a large amount of time, since it must be carried out under the immediate supervision of the surgeon, and that there is difficulty in finding persons who are capable of giving the skilled assistance required.

[1] New York Medical News, Jan. 2.

